# Pyrosequencing-Based Assays for Rapid Detection of HER2 and HER3 Mutations in Clinical Samples Uncover an E332E Mutation Affecting HER3 in Retroperitoneal Leiomyosarcoma

**DOI:** 10.3390/ijms160819447

**Published:** 2015-08-17

**Authors:** Paula González-Alonso, Cristina Chamizo, Víctor Moreno, Juan Madoz-Gúrpide, Nerea Carvajal, Lina Daoud, Sandra Zazo, Ester Martín-Aparicio, Ion Cristóbal, Raúl Rincón, Jesús García-Foncillas, Federico Rojo

**Affiliations:** 1 Group of Cancer Biomarkers, Pathology Department, Health Research Institute Fundación Jiménez Díaz (IIS-FJD, UAM), Avda. Reyes Católicos 2, 28040 Madrid, Spain; E-Mails: paula.galonso@fjd.es (P.G.-A.); chamizomunoz@gmail.com (C.C.); jmadoz@fjd.es (J.M.-G.); nerea.carvajal@fjd.es (N.C.); daoudlina@gmail.com (L.D.); szazo@fjd.es (S.Z.); ester.martin@fjd.es (E.M.-A.); rrincon23@gmail.com; (R.R.); 2 Translational Oncology Division, Oncohealth Institute, Fundación Jiménez Díaz Hospital, Avda. Reyes Católicos 2, 28040 Madrid, Spain; E-Mails: victor.moreno@fjd.es (V.M.); ion.cristobal@fjd.es (I.C.); jgfoncillas@fjd.es (J.G.-F.)

**Keywords:** HER2, HER3, cancer, pyrosequencing

## Abstract

Mutations in Human Epidermal Growth Factor Receptors (HER) are associated with poor prognosis of several types of solid tumors. Although HER-mutation detection methods are currently available, such as Next-Generation Sequencing (NGS), alternative pyrosequencing allow the rapid characterization of specific mutations. We developed specific PCR-based pyrosequencing assays for identification of most prevalent HER2 and HER3 mutations, including S310F/Y, R678Q, L755M/P/S/W, V777A/L/M, 774-776 insertion, and V842I mutations in HER2, as well as M91I, V104M/L, D297N/V/Y, and E332E/K mutations in HER3. We tested 85 Formalin Fixed and Paraffin Embbeded (FFPE) samples and we detected three HER2-V842I mutations in colorectal carcinoma (CRC), ovarian carcinoma, and pancreatic carcinoma patients, respectively, and a HER2-L755M mutation in a CRC specimen. We also determined the presence of a HER3-E332K mutation in an urothelial carcinoma sample, and two HER3-D297Y mutations, in both gastric adenocarcinoma and CRC specimens. The D297Y mutation was previously detected in breast and gastric tumors, but not in CRC. Moreover, we found a not-previously-described HER3-E332E synonymous mutation in a retroperitoneal leiomyosarcoma patient. The pyrosequencing assays presented here allow the detection and characterization of specific HER2 and HER3 mutations. These pyrosequencing assays might be implemented in routine diagnosis for molecular characterization of HER2/HER3 receptors as an alternative to complex NGS approaches.

## 1. Introduction

The protooncogenes Human Epidermal Growth Factor 2 (HER2) and Human Epidermal Growth Factor 3 (HER3) are members of the HER family of protein-tyrosine kinases. Ligand binding induces homodimerization or heterodimerization, and subsequent activation of Human Epidermal Growth Factor Receptors (HER) receptors that leads to cellular signaling. Whereas ligand-induced activation is a closely regulated process in normal cells, oncogenic activation of receptors may occur in cancer cells, either through overexpression, amplification, or activating mutations in the kinase domain or in the extracellular domain of the receptors [1]. ERBB receptors participate in the regulation of normal cell proliferation and survival [2]; however, their deregulation is associated with the development and progression of a wide variety of types of solid tumors [3]. Members of the ErbB family are important in etiology of human carcinomas, and particularly, HER2 is often overexpressed and Epidermal Growth Factor Receptor (EGFR) is mutated in different tumor types. During the last decade, HER2 and EGFR have become important biomarkers and targets of therapy for cancer patients [4,5]. On the other hand, scientists have also elucidated the critical role of the so-called codon bias, which consists on the effect of synonymous mutations in regulating gene expression and cellular function. That phenomenon affects diverse stages of mRNA transcription and protein synthesis [6]. In addition, Mazumder *et al*. have recently revealed that codon usage frequency determines the level of oncogene expression [7]. The authors have also deciphered that highly expressed oncogenes have rich guanine-cytosine content (GC-content), and that the oncogenicity of those genes positively correlates with GC-content at the third synonymous codon position [7]. Finally, Wheler *et al.* claim most of the identified genomic aberrations to predict the tumor sensitivity to some therapies, although they emphasize the need to revise the standard therapeutic pursuit of molecular alterations [8]. All of these data suggest that HER2 and HER3 mutational screening is indeed critical for predicting the potential clinical implication of HER2 and HER3 alterations, besides gene amplification, in most prevalent tumor types.

In recent years, the use of Next Generation Sequencing (NGS) has revealed the presence of HER2 somatic mutations in a variety of tumor tissues, such as brain, breast, colorectal, gastric, lung, and ovarian [9]. Moreover, Whole Exome Sequencing (WES) approaches have allowed the detection of HER3 somatic mutations in several tumor types, such as breast, and colon epithelial cells [10]. NGS and WES are especially effective for detection of HER2 and HER3 mutations in tumors. These are, however, laborious techniques for the routine clinical laboratory diagnosis, and require highly-qualified personal to perform the analysis of the results generated. Hence, a more accessible method would improve the efficiency of HER2 and HER3 molecular screening. As an alternative to laborious techniques, pyrosequencing is an already-established approach for the detection of previously identified mutations in formalin-fixed, paraffin-embedded (FFPE) tissue [11]. However, there is currently no research focused on the application of pyrosequencing on detection of HER aberrations in clinical diagnosis.

The aim of this study was to develop sensitive, rapid, and robust pyrosequencing assays for the detection and characterization of several of the prevalent HER2 and HER3 hotspot mutations in a variety of cancers. The pyrosequencing approach achieves immediate reliable results and pyrogram data analysis is more straightforward than NGS and WES data analysis. The results from this study suggests that the pyrosequencing assays described here might be implemented in routine diagnostics to identify HER2 and HER3 specific mutations in human cancers.

## 2. Results

**Figure 1:**
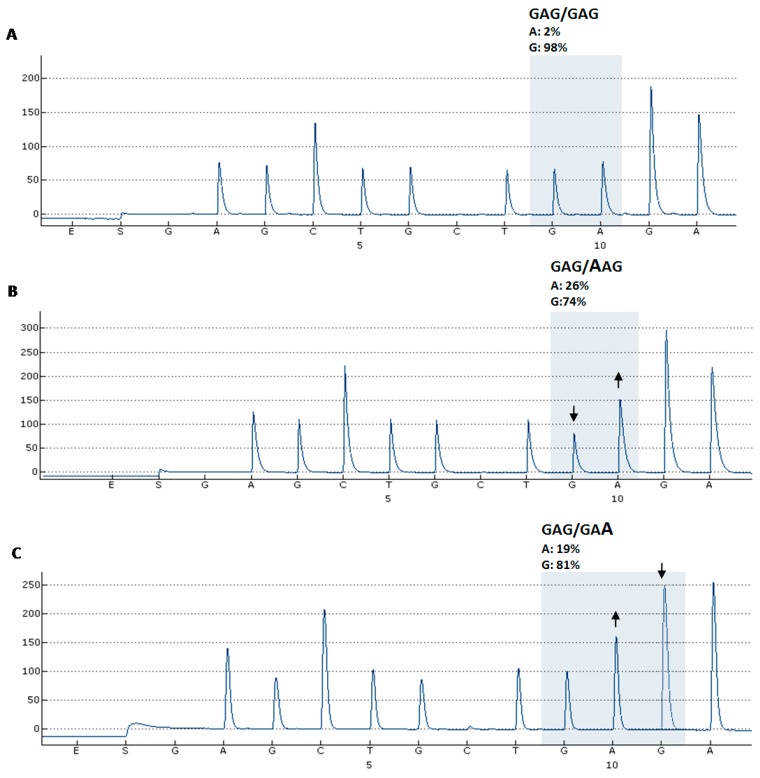
Representative pyrograms of the HER3 assay that detects E332K mutation and novel E332E synonymous mutation. (**A**–**C**) The nucleotide dispensation scheme during the E332 pyrosequencing assay is shown below each pyrogram. The ordinate axis displays signal intensity in arbitrary fluorescence units related to peak high. The shaded area of each pyrogram represents the nucleotides at the E332 somatic mutation site. The arrows show peak modifications corresponding to altered nucleotides at each somatic single nucleotide variant (sSNV) site. Allelic composition of the sSNVs and relative frequency of nucleotides adenine (**A**) *vs.* guanine (**G**) at a mutation site for a given position are indicated above each pyrogram. (**A**) Representative pyrogram trace of a tumor sample revealing a wild-type HER3 sequence at E332 site (GAG); (**B**) Pyrogram as displayed after an E332 pyrosequencing assay showing the classical E332K mutation, consisting of a substitution of an A nucleotide (upward-pointing arrow) in the place of a G nucleotide (downward-pointing arrow) at codon-first position; (**C**) Sequence analysis of the tumor sample harboring the novel E332E synonymous mutation, characterized as a substitution of an A nucleotide (upward-pointing arrow) in the place of a G nucleotide (downward-pointing arrow) at codon-third position.

We isolated nucleic acids from 86 specimens and 85 of the extracted samples were successfully PCR-amplified. PCR products were visualized in 2% agarose gel electrophoresis, which confirmed that each PCR assay lead to a singular PCR product corresponding to the expected size, the absence of primer-dimer formation, and the use of optimal physic and chemical PCR conditions (an additional file shows this in more detail (see Figure S1)). Then, successful HER2/HER3 mutational status was determined in 85 samples (Table 1). We detected seven missense mutations in several tumor type specimens by pyrosequencing. In particular, three V842I mutations in the HER2 tyrosine kinase domain were found in a variety of tissues, including colorectal carcinoma (CRC), cervix carcinoma (gynecologic carcinoma) and pancreatic carcinoma, respectively. We detected a L755M mutation in the HER2 tyrosine kinase domain of a CRC specimen. In addition, HER3 molecular analysis revealed three missense mutations in the HER3 furin-like cysteine-rich region: an E332K mutation was found in an urothelial carcinoma specimen, and two D297Y mutations were present in both gastric adenocarcinoma and CRC specimens. The pyrosequencing assay designed to detect E332K mutations in HER3 furin-like cysteine rich domain has also allowed us to detect a novel E332E synonymous mutation, which changes a GAG codon to a GAA codon (Figure 1).The E332E synonymous mutation was detected in a retroperitoneal leiomyosarcoma specimen (soft tissue sarcoma).

## 3. Discussion

HER mutations screening is critical for predicting the response to specific therapy with tyrosine kinase inhibitors (TKIs) in patients with a variety of cancers [12]. Frequently, FFPE samples are the only specimens available for diagnosis in routine clinical practice and, hence, molecular analyses are often hindered by the quality of DNA. Although NGS has been widely used to identify novel mutations, it consists of a high-throughput process that extends across millions of reactions simultaneously. These require considerable data analysis effort, making those technologies not suitable for clinical practice. In the present study, we demonstrate that pyrosequencing is a sensitive, robust and specific approach to accurately detect HER2 and HER3 mutations in DNA extracted from FFPE clinical samples. Nonetheless, the specimens might be inconclusive due to an insufficient amount or deficient state of the isolated material, with the consequent absence of nucleic acid amplification. This observation indicates that nucleic acid preservation in FFPE samples is a challenging step, and it therefore requires a cautious performance for the correct execution of the whole procedure described here.

The results presented here are consistent with the L755M and V842I mutations previously described at the cBioPortal for Cancer Genomics website [13] and The Cancer Genome Atlas (TCGA) [14,15]. Interestingly, the D297Y missense mutation has been previously described in breast and gastric cancer [16,17,18], yet this mutation has not been previously identified in CRC. As mentioned in the literature, an implication of this is the possibility that missense mutations might sensitize to some HER2/HER3 TKIs [4,12]. Though four synonymous mutations have been reported in the HER3 furin-like domain (RefSNP accession IDs: rs372292404, rs188312953, rs79638498, and rs37400753), to the best of our knowledge, the novel E332E synonymous mutation had neither been previously-described in the literature nor reported in any database of genetic variants [13,19,20,21]. The E332E synonymous mutation described here changes a GAG codon to a GAA codon. Recent research has reported that synonymous mutations may affect the splicing process, thereby altering the protein function [22,23]. Furthermore, there is some evidence that suggests that codon bias might affect oncogenicity of the target [7] and protein expression level [6], even though synonymous mutations lead to conserved amino acid sequence. Nonetheless, GAG has shown a high codon usage frequency among coding sequence of human oncogenes [7]. This combination of findings provides some support for the hypothesis that the GAA mutant codon might slightly reduce oncogenicity of HER3 protooncogene.

**Table 1:** Tumor type description and mutational profile of the samples included in the study.

*Tumor Type*	*Specimens*	*HER2*			*HER3*	
*Category*	*Sample size (n)*	*Mutants (n)*	*aa Change*	*wt/mutant codon*	*Mutants (n)*	*aa Change*
NSCLC	10	0	-	-	0	-
Breast Ca	2	0	-	-	0	-
CRC	34	1	L755M	TTG/ATG	1	D297Y
		1	V842I	GTA/ATA		
Bladder Urothelial Cell Ca	11	0	-	-	1	E332K
Epithelial Ovarian Ca	7	1	V842I	GTA/ATA	0	-
Endometrial Ca	3	0	-	-	0	-
Cervical Squamous Cell Ca	1	0	-	-	0	-
Gastric Ca	9	0	-	-	1	D297Y
Pancreatic Ca	2	1	V842I	GTA/ATA	0	-
Soft tissue sarcoma	3	0	-	-	1	E332E
RCC	2	0	-	-	0	-
Melanoma	1	0	-	-	0	-
Cutaneous SCC	1	0	-	-	0	-
Total	86	4			4	

Abbreviations: aa: amino acid; NSCLC: Non-small-cell lung carcinoma; Ca: Carcinoma; CRC: colorectal cancer; RCC: renal cell carcinoma; SCC: squamous cell carcinoma.

Sanger sequencing is the reference method used for the validation of mutations detected by other techniques, such as pyrosequencing. Nonetheless, an inherent limitation of direct sequencing by Sanger is the requirement of more than 300 base pairs (bp) in length of PCR products, which is necessary to avoid an oversaturation phenomenon which might lead to misidentification of the noise level with the specific signal [24]. Thereby, validation of the novel E332E synonymous mutation described here by Sanger sequencing would be notably challenging, due to the fragmented or low-quality DNA fragments present in FFPE samples. In addition, some authors have stated that Sanger sequencing validation consists of an arduous technique for the routine diagnosis, so that would not be necessary in the clinical practice [25].

## 4. Experimental Section

### 4.1. Clinical Samples

86 patients with a histological diagnosis of advanced solid tumors through at least one standard regimen of therapy at the Fundación Jiménez Díaz University Hospital (Madrid, Spain) were retrospectively selected for this study, as indicated in Table 1. The end of the follow up period was November 2014, and the median follow-up time was 33 months. Thus, previously diagnosed FFPE primary tumor biopsies from 45 male and 41 female patients were retrieved from the archive of the Hospital Biobank according to the Declaration of Helsinki principles and institutional review board policies. All participants provided written informed consent for the study. To further understand the clinical implications of HER2/HER3 mutations in human solid tumors, we performed HER2 and HER3 mutational profiling in genomic material from all specimens included in the study.

### 4.2. Nucleic Acid Extraction and cDNA Synthesis

Tumor samples containing at least 80% tumor nuclei were deparaffined in xylene followed by ethanol washing. Then, either genomic DNA (gDNA), or total RNA were extracted using the QIAamp DNA FFPE Tissue kit or the RNeasy FFPE kit (Qiagen, Hilden, Germany), respectively from 5 × 10 μm sections of FFPE tissue. gDNA and RNA extractions were performed according to the manufacturer’s recommendations. The quantity and purity of extracted gDNA and RNA were estimated by NanoDrop (ND-2000 UV-Vis Spectrophotometer; Nanodrop Technologies Inc., Walthman, MA, USA). cDNA was produced using the Universal Transcriptor cDNA synthesis kit (Roche Diagnostics, Barcelona, Spain) according to the manufacturer’s recommendations.

### 4.3. PCR Primers Design and PCR Amplification

**Figure 2:**
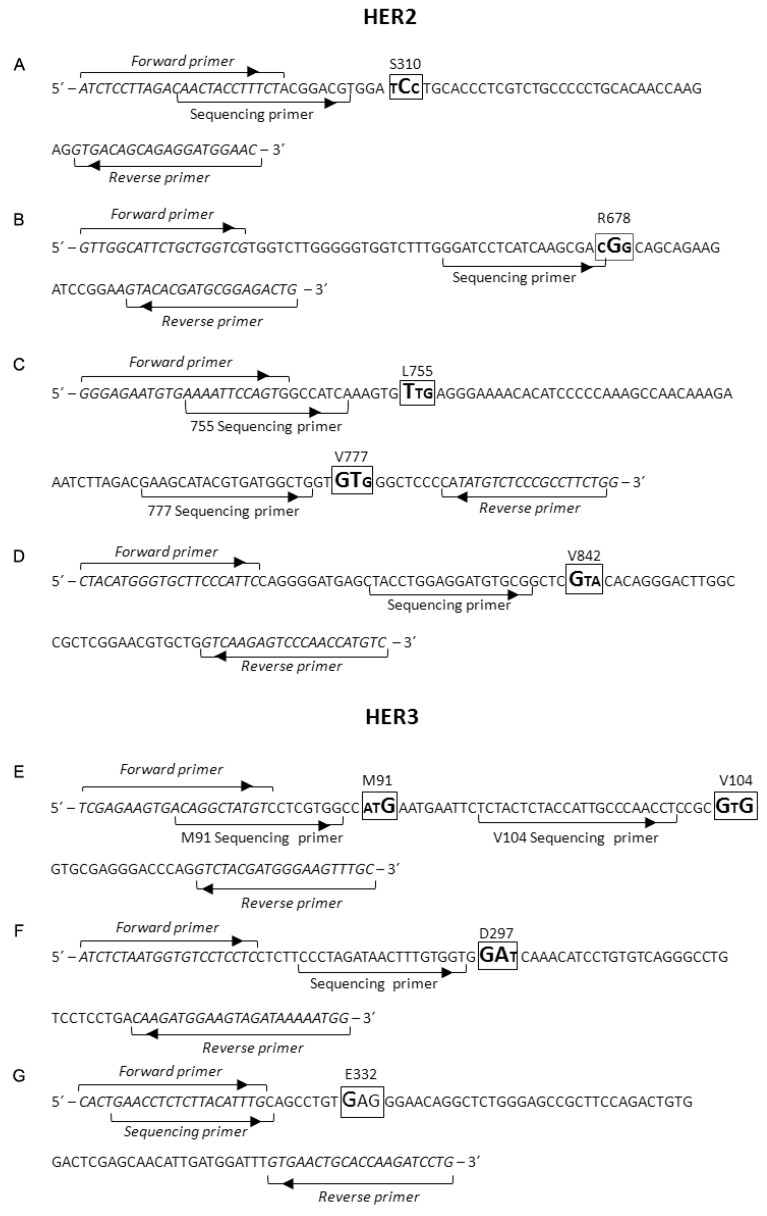
Pyrosequencing assay maps. These show PCR sequence and target mutations, as well as primer designs for PCR amplification and pyrosequencing for HER2 and HER3 mutation analysis. Arrows indicate the specific hybridization site for both PCR and sequencing primers, and the 5′ to 3′ direction for DNA-polymerase amplification. All reverse primers for PCR amplification are 5′-biotinylated. (**A**–**D**) Primer designs for HER2 assays (**A**) S310, (**B**) R678, (**C**) L755 and V777, (**D**) V842; (**E**–**G**) Primer designs for HER3 assays (**E**) M91 and V104, (**F**) D297, and (**G**) E332.

PCR specific primers are described in Figure 2, and were designed using the Entrez Global Query Cross-Database Search System and the Basic Local Alignment Search Tool (BLAST) of the National Centre for Biotechnology Information (NCBI) website [26], the Lasergene sequence analysis software (DNAStar, Madison, WI, USA), and the ProbeFinder software (Roche, Mannheim, Germany). Specific amplification primers were designed for HER2 amplification [GenBank: NG_007503.1, NM_004448.3, and NM_001005862] and HER3 amplification [GenBank: NG_011529.1, NM_001982.3, and NM_001005915.1]. Since 755 and 777 sites are located in different exons of HER2, 755-777 PCR was performed with cDNA, thus a single PCR allowed characterization of two mutation sites, as well as the characterization of 777 insertions. Primer design was performed according to the following conditions: (i) the forward (Fw) and reverse (Rv) primers are located in different regions; gDNA-PCR primers spanned exon/introns junctions to ensure gDNA amplification; cDNA-PCR primers spanned exon junctions or located in different exons to avoid gDNA amplification; (ii) the primer composition had an optimal A/T:G/C ratio in order to achieve an annealing temperature that ranges from 58 to 64 °C; (iii) the primers showed absence of secondary structures formation, such as hairpin, self-dimer and dimer-primer; (iv) the BLASTN unspecific e-value was at least one order of magnitude superior to the specific e-value; (v) the size of the amplicon product ranged from 90 to 150 base pairs (bp) in length.

PCR analyses were carried out on a Verity Thermal Cycler (Applied Biosystems) and were performed using 50 ng of template (cDNA or gDNA), 0.2 μM of each amplification primer, 5 U/μL of Fast Start Taq polymerase (Roche), 1× buffer with Mg^2+^ at a final concentration of 2 mM, deoxyribonucleotydes (dNTPs) at a final concentration of 0.2 mM, and sterile nuclease free water to a final reaction volume of 20 μL. The cycling conditions were identical for all amplifications and consisted of two steps that included 10 min an initial incubation at 95 °C, followed by 45 cycles of 95 °C for 10 s, and 60 °C for 30 s.

### 4.4. Electrophoresis

PCR products were visualized in 2% agarose gel electrophoresis in order to assess the PCR success and specificity, and the proper size of the resulting product (see Supplementary Material). Resulting products from either effective 755, yet ineffective 777 pyrosequencing assays, or ineffective 755 and 777 pyrosequencing assays, were visualized by 2% agarose gel in order to determine the presence of a 777 insertion or the ineffectivity of both 755 and 777 specific PCR.

### 4.5. Pyrosequencing Primers Design and Pyrosequencing Assay

PCR products were processed according to the manufacturer’s instructions previous to pyrosequencing assay on a PyroMark Q24 instrument (Qiagen, Hilden, Germany). We examined the mutational status of HER2 in exons 12, 21, 23, 24, and 25 [GenBank: NG_007503.1], and the status of HER3 in exons 3, 8 and 9 [GenBank: NG_011529.1], in 86 tumor samples by pyrosequencing assay with the specific primers indicated in the Figure 2.

The amplicon-specific pyrosequencing primers were designed according to the following conditions: the annealing distance to the mutation ranged from none to seven nucleotides; the primers showed absence of secondary structures formation; and the primer composition had an optimal A/T:G/C ratio in order to achieve an annealing temperature under 60 °C. Pyrosequencing primers were used at a final concentration of 0.3 μM in annealing buffer. The nucleotide dispensation order (NDOs) for each assay was optimized to obtain the best result in the study of each mutation, according to the sequence to analyze, and they are described in Table 2. Then, we analyzed the obtained pyrogram data to evaluate detected HER2/HER3 mutations by using Pyromark software (Qiagen).

**Table 2:** Nucleotide dispensation orders of HER2 and HER3 assays.

Target	Assay	NDO
**HER2**	S310	GTGATCTGTCACT
	R678	TAGCAGTCAGAG
	L755	GAGTCGATCGAG
	V777	CGATGCATGCT
	V842	AGCTCAGTACGACAG
**HER3**	M91	TCACTCGATCGA
	V104	GCGCTGACTACGTAGC
	D297	CGTATCGACA
	E332	GAGCTGCTGAGA

Abbreviations: NDO: Nucleotide Dispensation Order.

## 5. Conclusions

Our approach lacked a homogeneous, representative, and sample-sized set of patient cases to establish the prognostic and/or predictive value for the hotspot mutations described here. For this purpose, it would be necessary to examine a larger number of randomly-selected samples for every type of tumor. Still, our results suggest that the pyrosequencing assays presented here might be a promising approach in molecular characterization of classical and uncommon HER2/HER3 mutations. Pyrosequencing allows parallel processing of samples and can be easily automated. Even though further studies would be necessary to establish the detection limit for these original pyrosequencing assays, they might be implemented in routine diagnosis for HER2 and HER3 reliable mutation tests as an alternative to currently available laborious methods.

## Supplementary Materials

Supplementary materials can be found at http://www.mdpi.com/1422-0067/16/08/19447/s1.

## References

[1] Hynes N.E., Lane H.A. (2005). ErbB receptors and cancer: The complexity of targeted inhibitors. Nat. Rev. Cancer.

[2] Burgess A.W., Cho H.S., Eigenbrot C., Ferguson K.M., Garrett T.P., Leahy D.J., Lemmon M.A., Sliwkowski M.X., Ward C.W., Yokoyama S. (2003). An open-and-shut case? Recent insights into the activation of EGF/ErbB receptors. Mol. Cell.

[3] Baselga J., Swain S.M. (2009). Novel anticancer targets: Revisiting ErbB2 and discovering ErbB3. Nat. Rev. Cancer.

[4] Bose R., Kavuri S.M., Searleman A.C., Shen W., Shen D., Koboldt D.C., Monsey J., Goel N., Aronson A.B., Li S. (2013). Activating HER2 mutations in HER2 gene amplification negative breast cancer. Cancer Discov..

[5] Sholl L.M., Xiao Y., Joshi V., Yeap B.Y., Cioffredi L.A., Jackman D.M., Lee C., Janne P.A., Lindeman N.I. (2010). *EGFR* mutation is a better predictor of response to tyrosine kinase inhibitors in non-small cell lung carcinoma than FISH, CISH, and immunohistochemistry. Am. J. Clin. Pathol..

[6] Plotkin J.B., Kudla G. (2011). Synonymous but not the same: The causes and consequences of codon bias. Nat. Rev. Genet..

[7] Mazumder T.H., Chakraborty S., Paul P. (2014). A cross talk between codon usage bias in human oncogenes. Bioinformation.

[8] Wheler J.J., Parker B.A., Lee J.J., Atkins J.T., Janku F., Tsimberidou A.M., Zinner R., Subbiah V., Fu S., Schwab R. (2014). Unique molecular signatures as a hallmark of patients with metastatic breast cancer: Implications for current treatment paradigms. Oncotarget.

[9] Herter-Sprie G.S., Greulich H., Wong K.K. (2013). Activating mutations in ErbB2 and their impact on diagnostics and treatment. Front. Oncol..

[10] Jaiswal B.S., Kljavin N.M., Stawiski E.W., Chan E., Parikh C., Durinck S., Chaudhuri S., Pujara K., Guillory J., Edgar K.A. (2013). Oncogenic ErbB3 mutations in human cancers. Cancer Cell.

[11] Sundstrom M., Edlund K., Lindell M., Glimelius B., Birgisson H., Micke P., Botling J. (2010). *KRAS* analysis in colorectal carcinoma: Analytical aspects of pyrosequencing and allele-specific PCR in clinical practice. BMC Cancer.

[12] Gandhi L., Bahleda R., Tolaney S.M., Kwak E.L., Cleary J.M., Pandya S.S., Hollebecque A., Abbas R., Ananthakrishnan R., Berkenblit A. (2014). Phase I study of neratinib in combination with temsirolimus in patients with human epidermal growth factor receptor 2-dependent and other solid tumors. J. Clin. Oncol..

[13] cBioPortal for cancer genomics. http://www.cbioportal.org/.

[14] Cerami E., Gao J., Dogrusoz U., Gross B.E., Sumer S.O., Aksoy B.A., Jacobsen A., Byrne C.J., Heuer M.L., Larsson E. (2012). The cBio cancer genomics portal: An open platform for exploring multidimensional cancer genomics data. Cancer Discov..

[15] Gao J., Aksoy B.A., Dogrusoz U., Dresdner G., Gross B., Sumer S.O., Sun Y., Jacobsen A., Sinha R., Larsson E. (2013). Integrative analysis of complex cancer genomics and clinical profiles using the cBioPortal. Sci. Signal..

[16] Stephens P.J., Tarpey P.S., Davies H., van Loo P., Greenman C., Wedge D.C., Nik-Zainal S., Martin S., Varela I., Bignell G.R. (2012). The landscape of cancer genes and mutational processes in breast cancer. Nature.

[17] Cancer Genome Atlas Network (2012). Comprehensive molecular portraits of human breast tumours. Nature.

[18] Holbrook J.D., Parker J.S., Gallagher K.T., Halsey W.S., Hughes A.M., Weigman V.J., Lebowitz P.F., Kumar R. (2011). Deep sequencing of gastric carcinoma reveals somatic mutations relevant to personalized medicine. J. Transl. Med..

[19] Cosmic: Catalogue of somatic mutations in cancer. http://cancer.sanger.ac.uk/cancergenome/projects/cosmic.

[20] NCBI (Nacional center for biotechnology information) dbSNP: Database of single nucleotide polymorphisms (SNPs). http://www.ncbi.nlm.nih.gov/snp.

[21] UniProt: Universal protein resource. http://www.uniprot.org/uniprot/P21860.

[22] Zheng S., Kim H., Verhaak R.G. (2014). Silent mutations make some noise. Cell.

[23] Supek F., Minana B., Valcarcel J., Gabaldon T., Lehner B. (2014). Synonymous mutations frequently act as driver mutations in human cancers. Cell.

[24] Prober J.M., Trainor G.L., Dam R.J., Hobbs F.W., Robertson C.W., Zagursky R.J., Cocuzza A.J., Jensen M.A., Baumeister K. (1987). A system for rapid DNA sequencing with fluorescent chain-terminating dideoxynucleotides. Science.

[25] Righi L., Cuccurullo A., Vatrano S., Cappia S., Giachino D., de Giuli P., Ardine M., Novello S., Volante M., Scagliotti G.V. (2013). Detection and characterization of classical and “uncommon” exon 19 epidermal growth factor receptor mutations in lung cancer by pyrosequencing. BMC Cancer.

[26] NCBI Nacional center for biotechnology information. http://www.ncbi.nlm.nih.gov/.

