# Supplementary material for: Pyrosequencing-Based Assays for Rapid Detection of HER2 and HER3 Mutations in Clinical Samples Uncover an E332E Mutation Affecting HER3 in Retroperitoneal Leiomyosarcoma
Source: Int J Mol Sci. 2015 Aug 17;16(8):19447–57. doi: 10.3390/ijms160819447 (PMC4581306; doi:10.3390/ijms160819447)
Supplement: Supplementary file 1 [file ijms-16-19447-s001.pdf]

Supplementary Information

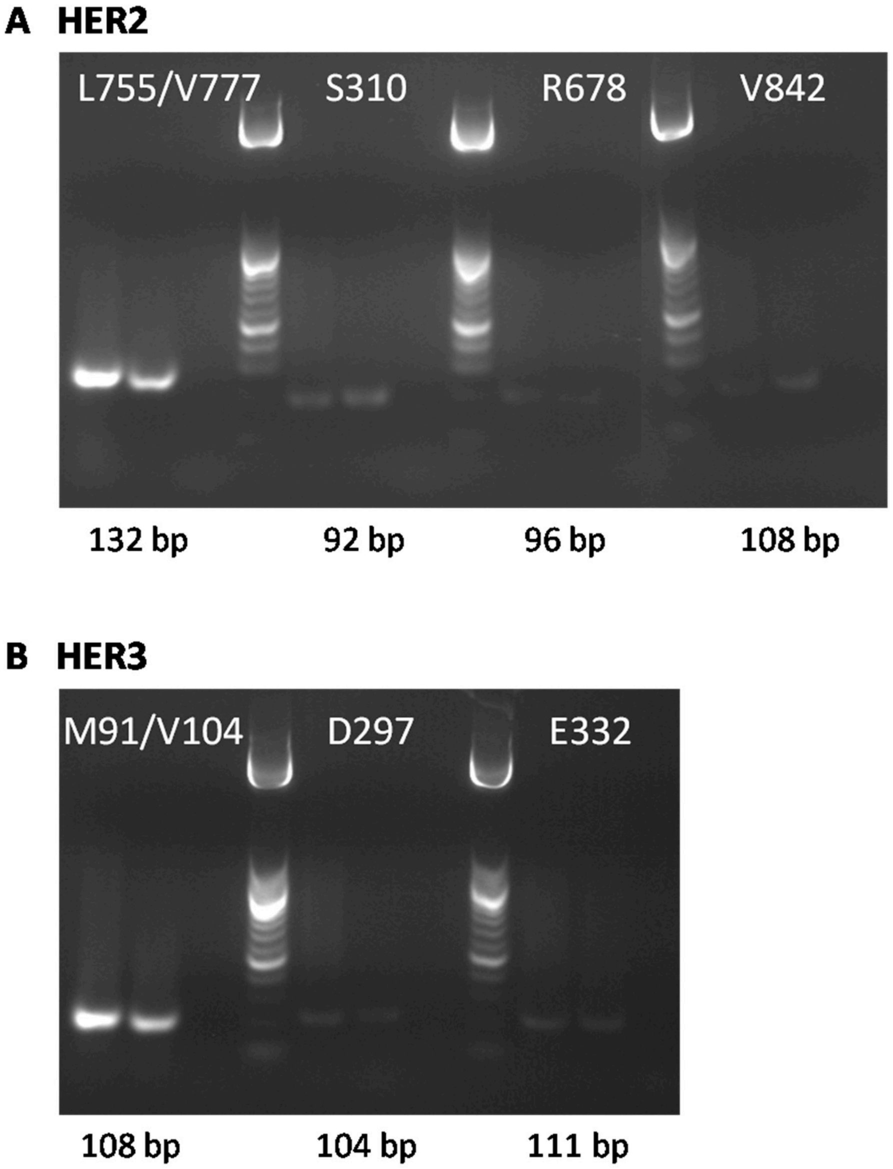

Figure S1. 2% agarose gel electrophoresis shows PCR success and primer specificity. (**A**,**B**) Molecular Weight Marker XIII (Roche) lanes separate different PCR assay areas. Lanes within each PCR assay show loaded PCR products from tumor sample, MSA (C+), and NTC, respectively. Each PCR assay shows a unique PCR product corresponding to the expected size. NTC lanes showing undetected signal indicate the absence of primer-dimer formation. Results obtained within all lanes reveal optimally adjusted physic and chemical PCR conditions. (**A**) HER2 PCR amplification assays; (**B**) HER3 PCR amplification assays. Abbreviations: C+: positive control. NTC: Non-Template Control.
